# SC75741, A Novel c-Abl Inhibitor, Promotes the Clearance of TDP25 Aggregates *via* ATG5-Dependent Autophagy Pathway

**DOI:** 10.3389/fphar.2021.741219

**Published:** 2021-10-29

**Authors:** Dongheng Zhou, Huanhuan Yan, Shuying Yang, Yuhong Zhang, Xiaoyan Xu, Xufeng Cen, Kai Lei, Hongguang Xia

**Affiliations:** ^1^ Department of Biochemistry and Research Center of Clinical Pharmacy of The First Affiliated Hospital, Zhejiang University School of Medicine, Hangzhou, China; ^2^ Liangzhu Laboratory, Zhejiang University Medical Center, Hangzhou, China; ^3^ Westlake Laboratory of Life Sciences and Biomedicine, Key Laboratory of Growth Regulation and Translational Research of Zhejiang Province, School of Life Sciences, Westlake University, Hangzhou, China; ^4^ Institute of Biology, Westlake Institute for Advanced Study, Hangzhou, China

**Keywords:** autophagy, SC75741, TDP43, TDP25, c-Abl, ALS, TFEB

## Abstract

Abnormal accumulation of TDP43-related mutant proteins in the cytoplasm causes amyotrophic lateral sclerosis (ALS). Herein, unbiased drug screening approaches showed that SC75741, a multi-target inhibitor, inhibited inflammation-induced aggregation by inhibiting NF-κB and also degraded already aggregated proteins by inhibiting c-Abl mediated autophagy-lysosomal pathway. We delineate the mechanism that SC75741 could markedly enhance TFEB nuclear translocation by an mTORC1-independent TFEB regulatory pathway. In addition, SC75741 enhanced the interaction between p62 with TDP25 and LC3C, thus promoting TDP25 degradation. Taken together, these findings show that SC75741 has beneficial neuroprotective effects in ALS. Our study elucidates that dual-targeted inhibition of c-Abl and NF-κB may be a potential treatment for TDP43 proteinopathies and ALS.

## Introduction

Amyotrophic lateral sclerosis (ALS) is a fatal neurodegenerative disease associated with progressive muscle denervation, weakness, atrophy, spasticity, paralysis, and eventually death (Mitchell and Borasio, 2007). About 90% of ALS cases are sporadic (sALS), and only 10% are familial ALS (fALS) (Leblond et al., 2014; Trojsi et al., 2020). In the past 2 decades, at least 25 genes are closely related to fALS and sALS (Brown and Al-Chalabi, 2017). Currently, there are only two FDA-approved drugs (Riluzole and Edaravone) for ALS treatment (Sojka et al., 1997; Rothstein, 2017). However, these drugs cannot prevent or reverse neurodegenerative conditions (Jaiswal, 2019). Therefore, it is necessary to find a novel therapeutic strategy for ALS treatment.

TDP43, a DNA-RNA binding protein encoded by the TARDBP gene, was first identified in 2006 as the key aggregate component found in the brains of ALS patients (Neumann et al., 2006; Buratti and Baralle, 2008; Wang et al., 2008). Notably, about 97% of ALS patients have TDP43-related mutations and only 3% of SOD1 and FUS mutations (Mackenzie et al., 2007; Ling et al., 2013). The loss of TDP43 nuclear function and the gain of cytoplasmic toxicity causes both familial and sporadic ALS (Barmada et al., 2010; Cascella et al., 2016). Genetic mutations, inflammations, and other factors lead to post-translational modifications of TDP43, which are aberrantly hyperphosphorylated, ubiquitinated, and cleaved to generate C-terminal fragments of 35 and 25 kDa (Zhang et al., 2009; Caccamo et al., 2013; Cicardi et al., 2018; Palomo et al., 2019). TDP25 can also be mislocated in the cytoplasm to form toxic, insoluble, ubiquitin- and phospho-positive aggregates (Gregory et al., 2012; Kitamura et al., 2018). To date, there is no effective drug to clear TDP43-related aggregates.

C-Abl, a non-receptor tyrosine kinase, was originally discovered as an oncogene, which can be fused with B cell receptors to cause chronic myeloid leukemia (Ren, 2005). Since its discovery, it has been found to be involved in a wide variety of cellular processes including signal transduction, cell adhesion, cell cycle, oxidative stress and DNA damage (Yuan et al., 1999; Wagner et al., 2008; Cabigas et al., 2012). In recent years, c-Abl has been found to play an important role in neurodegenerative diseases (Schlatterer et al., 2011; Tanabe et al., 2014; Rojas et al., 2015). c-Abl has been shown to phosphorylate tau protein to promote neurofibrillary tangles and form aggregates in AD (Tremblay et al., 2010). In addition, the level of c-Abl in the brain of APP/Swe mice is higher than control mice, when treated with the c-Abl inhibitor STI571, the level of phosphorylated tau of APP mice decreased significantly (Cancino et al., 2011). The aggregation of α-synuclein in the brain has been proved to be the main cause of Parkinson’s disease. Nilotinib, a c-Abl inhibitor, has been reported to promote the clearance of α-syn aggregates and alleviate PD pathology (Hebron et al., 2013). Interestingly, multiple studies have shown phosphorylated c-Abl activation in human ALS motor cortex. Inhibition or knockdown of c-Abl in ALS patient iPSC-derived motor neurons promoted motor neuron survival (Imamura et al., 2017). Therefore, c-Abl inhibitors are a potential strategy for the treatment of neurodegenerative diseases.

Autophagy-lysosomal pathway (ALP) and the ubiquitin-proteasome system (UPS) are the protein degradation pathways (Korolchuk et al., 2010; Ciechanover and Kwon, 2015; Kliosnky, 2016; Ji and Kwon, 2017). Over 80% of normal and abnormal intracellular proteins are degraded *via* the UPS pathway. However, ubiquitinated protein aggregates cause UPS impairment which prevents their recognition and degradation in several neurodegenerative diseases (Huang and Figueiredo-Pereira, 2010; Hipp et al., 2014; Kumar et al., 2020). Autophagy is an essential degradation pathway involved in the clearance of abnormal protein aggregates when the UPS is dysfunctional. Therefore, the discovery of an autophagy modulator can potentially degrade TDP43-related aggregates, thus treating ALS.

In this study, 2110 FDA-approved drugs and drug candidates were screened and identified SC75741 as a novel TDP25 degradation agent. We demonstrate that SC75741 promoted the degradation of TDP-43-related protein aggregation and reduced the inflammation-induced aggregation by inhibiting NF-κB pathway. SC75741, as a new c-Abl inhibitor, enhance autophagy activity in ATG5-dependent manner and activate TFEB nuclear translocation in mTORC1-independent manner. SC75741 also enhanced the interaction between p62 with TDP25 and LC3C, promoted mitigation of TDP25 aggregation by activation of ALP.

## Materials and Methods

### Cell Culture

HEK293WT, HEK293p62^−/−^, HEK293ATG5^−/−^, HelaWT, Hela ATG8^−/−^ and N2A cells were grown in DMEM medium (Hyclone, with L-glutamine, with 4.5 g/L glucose, without pyruvate); H4 cells were grown in DMEM medium (Hyclone, with L-glutamine, with 4.5 g/L glucose, with pyruvate); SH-SY5Y cells were grown in MEM medium (Hyclone, with L-glutamine, with earle’s balanced salts); These media were supplemented with 10% FBS (Gibco TM), 1% Penicillin/Streptomycin (Gibco TM). Doxycycline-inducible H4GT25, SH-SY5YGT25 and SH-SY5YKT25 stable cell lines were generated by co-transfecting HP138-GFP-TDP25 (HP138-Keima-TDP25) and HP216 plasmids (a gift from Dr. Hui Yang) into H4 and SH-SY5Y cells using Lipofectamine TM 2000 (Invitrogen TM) and selected with 10 μg/ml puromycin (Sangon® Biotech). All cells were cultured at 37°C with 5% CO_2_.

### Reagents and Antibody Generation

The chemicals and their sources are as follows: Doxycycline (#A603456), Puromycin (#A606719), NH4Cl (#A501569) from Sangon® Biotech; MG132 (#S2619), Bafilomycin A1 (#S1413), E-64D (#S7393), Leupeptin hemisulfate (#S7380) from SelleckChem; Arsenite (#S7400) from Sigma; SC75741 (#T6661), Ibudilast (#T2137), Ouabain (#T1318), Imatinib (#T6230), PP-121 (#T2415), JSH-23 (#T1930), CAPE (#T6429) from Topscience, Inc. (Shanghai, China); Anti-Flag (DYKDDDDK) Affinity Gel (#B23102) and Anti-HA magnetic beads (#B26202) were purchased from Bimake; Anti-GFP beads (#SM03801) was purchased from Smartlifesciences; Lipofectamine TM 2000 (#1901433) and Lipofectamine TM 3000 (#2067450) were from Invitrogen. The following antibodies were used in this study: β-tubulin (#M1305-2), Flag-Tag (#M1403-2), HA-Tag (#0906-1), GFP-Tag (#ET1607-31) from HuaAn Biotechnology; c-Abl (#2862), phospho-c-Abl (Tyr245) (#2861), TFEB (#37785), NF-κB (#8242), phospho-NF-κB (#5733) from Cell Signaling Technology®; LC3C (#18726-1-AP), p62 (#18420-1-AP) from Proteintech TM; LC3C (#A8295), Lamp1 (#A16894), TFEB (#A7311) from ABclonal; The secondary antibodies for western blotting were used: Goat anti-Mouse IgG (H + L) (#31430, Thermo Fisher Scientific), Goat anti-Rabbit IgG (H + L) (#31460, Thermo Fisher Scientific); The fluorescent secondary antibodies for immunofluorescence were used: goat anti-rabbit Alexa Fluor 546 (#A-11010), goat anti-mouse Alexa Fluor 405 (#A-31553) from Thermo Fisher Scientific.

### High Throughput Screening

All FDA approved drugs and bioactive compounds were commercially purchased from Topscience, Inc. (Shanghai, China). Drug in the high-throughput screening was 10 μg/ml. MG132 (1 μM) treatment was used as positive control to induce aggregate formation, ibudilast (10 μM) was used as a negative control to promote aggregate degradation. High-throughput screening was done using Biotek Cytation® 3 system excitation at 488 nm and emission at 510 nm. Every plate has negative control (MG132) and positive control (ibudilast), every plate has three replicates, and the screen Z-factor of every plate was >0.5. There are three screenings. In the first screening, 1 × 10^5^ H4GT25 cells were pretreated with 1 μg/ml DOX (doxycycline) for 24 h, incubated with 2110 FDA approved drugs and bioactive compounds for 24 h. MG132 (1 μM) treatment was used as negative control to induce aggregate formation, ibudilast (10 μM) was used as a positive control to promote aggregate degradation. The GFP-TDP25 fluorescence was analyzed by fluorescence microscopy and compared with fluorescence of cells treated with DMSO. A total of 178 small molecules with fluorescence intensity below 50% were obtained as candidate compounds. In the second screening, 1 × 10^5^ H4GT25 cells were pretreated with 1 μg/ml DOX for 24h, treated with aresnite (1 h) or MG132 (12 h), incubated with 178 hits for 24h, and the GFP-TDP25 fluorescence was analyzed by fluorescence microscopy and compared with fluorescence of cells treated with DMSO. A total of 2 small molecules with fluorescence intensity below 50% were obtained as candidate compounds. In the third screening, 1 × 10^5^ H4GT25 cells were pretreated with 1 μg/ml DOX for 24 h, treated with aresnite (1 h) or MG132 (12 h), and treated with Ibudilast (10 μM), SC75741 (10 μM) or ouabain (10 μM) for another 24 h, analyzed by fluorescence microscopy and cell viability. Cell viability was detected using CellTiter-Glo® Luminescent Cell Viability Assay (#G7573 Promega) according to manufacturer’s instructions. The average fluorescence intensity of GFP-TDP25 aggregates was used to analyze the statistics of the three screening rounds. The aggregates with an average fluorescence intensity >5,000 were counted. Statistical analysis was performed with Gen5™ 3.02 software.

### Immunoblotting

Cell lysates or immunoprecipitation samples were added SDS-PAGE 2Х loading buffer and heated at 100°C for 10 min, subjected to 10–15% SDS-PAGE and then transferred onto PVDF membranes for 0.5–1 h at 0.2 A with semi-dry transfer system of BioRad. Membranes were blocked in PBST buffer containing 10% (w/v) skimmed milk for 1 h and probed with the indicated antibodies in PBST containing 5% (w/v) BSA at 4°C overnight. Detection was performed using HRP-conjugated secondary antibodies and chemiluminescence reagents (#4AW001-500, 4 A Biotech Co.).

### Cell Viability

H4GT25 cells were pretreated with 1 μg/ml DOX for 24 h, treated with aresnite (1 h) or MG132 (12 h), and treated with ibudilast (10 μM), SC75741 (10 μM) or ouabain (10 μM) for another 24 h, cell viability was detected using CellTiter-Glo® Luminescent Cell Viability Assay (#G7573 Promega) according to manufacturer’s instructions.

### Solubility and Insolubility Protein Analysis

H4GT25 cells were pretreated with 1 μg/ml DOX for 24 h, treated with or wihtout 5 μM SC75741 for another 24 h, cells were washed twice with phosphate-buffered saline, lysed in cold RIPA buffer (1% Triton X-100,100 mM Tris-HCl-pH8.8, 100 mM NaCl, 0.5 mM EDTA, Protease inhibitor cocktail (Bimake, added fresh)), and sonicated. Cell lysates were cleared by centrifugation at 100,000 g for 30 min at 4°C to generate the RIPA-soluble samples. The supernatants collected from the first centrifugation was analyzed. Then, the resulting pellets were washed twice with RIPA buffer. RIPA-insoluble pellets were then extracted with urea buffer [7 M urea, 2 M thiourea, 4% CHAPS, 30 mM Tris, pH 8.5, Protease inhibitor cocktail (Bimake, added fresh)], sonicated, and centrifuged at 100,000 g for 30 min at 22°C.

### Nuclei-Cytoplasmic Fractions

H4GT25 cells were pretreated with 1 μg/ml DOX for 24 h, treated with or wihtout 5 μM SC75741 for another 24 h, cells were washed twice with phosphate-buffered saline, Lyse cells with 0.5 ml of 0.5% Triton X-100 lysis buffer (50 mM Tris-HCl (pH 7.5), 0.5% Triton X-100, 137.5 mM NaCl, 10% Glycerol, 5 mM ethylenediaminetetraacetic acid (EDTA) and protease inhibitor cocktail (Bimake, added fresh)) for 15 min in ice, shaking gently. Cell lysates were cleared by centrifugation at 13,000 rpm for 15 min at 4°C to generate the cytosolic and membrane fractions. The supernatants collected from the first centrifugation were analyzed. Then, the resulting pellets were washed twice with lysis buffer. Resuspend the pellet in 0.1 ml of lysis buffer, supplemented with 0.5% sodium dodecyl sulfate (SDS), and sonicate in cold room three times for 3 s at low output to shear genomic DNA. Cell lysates were cleared by centrifugation at 13,000 rpm for 15 min at 4°C to generate the nuclear fractions.

### Quantitative Real-Time PCR

Total RNA from H4GT25 cells was extracted using an RNA isolation Kit (Vazyme), and reverse-transcribed to cDNA using the HiScript III first Strand cDNA Synthesis Kit (Vazyme). Real-time PCR assays were performed using B818-480II (Rocher Applied Science). The oligonucleotides used were as follows: GAPDH; fw: 5′-att​gtt​cgt​cat​ggg​tgt​gaa-3′, rev: 5′-agg​ggt​gct​aag​cag​ttg​gt-3′, DPP7; fw: 5′-gat​tcg​gag​gaa​cct​gag​tg-3′, rev: 5′-cgg​aag​cag​gat​ctt​ctg​g-3′, CTSD; fw: 5′-ctt​cga​caa​cct​gat​gca​gc-3′, rev: 5′-tac​ttg​gag​tct​gtg​cca​cc-3′, MCOLN1; fw: 5′-gag​tcc​ctg​cga​caa​gtt​tc-3′, rev: 5′-tgt​tct​ctt​ccc​gga​atg​tc-3′, LAMP1; fw: 5′-cca​act​tct​ctg​ctg​cct​tc-3′, rev: 5′-agc​aat​caa​cga​gac​tgg​gg-3’. CTSF; fw: 5′-aca​gag​gag​gag​ttc​cgc​act​a-3′, rev: 5′-gct​tgc​ttc​atc​ttg​ttg​cca-3′, ATP6V0E1; fw: 5′-cat​tgt​gat​gag​cgt​gtt​ctg​g-3′, rev: 5′-aac​tcc​ccg​gtt​agg​acc​ctt​a-3′, SQSTM1; fw: 5′-cag​gtc​ttc​caa​ggt​tga​gg-3′, rev: 5′-ata​aaa​aca​cgg​cca​ttt​gc-3′, Map1LC3; fw: 5′- agc​gct​aca​agg​gtg​aga​a-3′, rev: 5′-gtt​cac​cag​cag​gaa​gaa​g-3’.

### Fluorescence Microscopy

Cells were cultured in 12-well plates on circular glass coverslips and washed twice with PBS and fixed with 4% paraformaldehyde for 30 min at room temperature. Cells were blocked with blocking buffer (5% FBS, 0.1%Triton X-100, PBS) at room temperature for 1 h, and incubated with primary antibodies overnight at 4°C. After washing with PBS, secondary antibodies were incubated at room temperature for 1 h. All samples were examined with an inverted FV3000 confocal microscope system using a 60 ×1.42-NA oil objective. All images were taken at room temperature and analyzed with FV10-ASW 4.0a Viewer.

### Live Cell Imaging 3D Microscopy Analysis

H4GT25 cells were grown in confocal dishes, pretreated with 1 μg/ml DOX for 24 h, treated with 5 μM SC75741 and using Nanolive Fluo-3D Cell Explorer® (Nanolive) microscope to explore aggregates change for a time-lapse of 720 min every 30s (60× magnification and depth of field 30 μm). images were exported using Steve software v.2.6® (Nanolive).

### Keima-TDP25 Assay

SH-SY5YKT25 cells were transfected with HP138-Keima-TDP25, treated with 1 μg/ml DOX for 24 h, treated with or without SC75741 (5 μM) for another 24 h. Imaging was done using Biotek Cytation® 3 system excitation at 469 nm/586 nm and emission at 620 nm. Autophagy levels were estimated by the fraction of cells that were fluorescent upon excitation at 469 and 586 nm using Gen5™ software. Subsequently, the Keima-green/Keima-red ratio and Keima-red/Keima-green ratio were calculated. For living-cell colocalization imaging, SH-SY5YKT25 stable cells were grown in confocal dishes and stained with LysoTracker™ Green DND-26 (#L7526, Thermo Fisher Scientific, Ltd.) and imaged with FV3000 confocal microscope system at 37°C with 5% CO_2_.

### Quantification and Statistical Analysis

High throughput screening analysis: The average fluorescence intensity of GFP-TDP25 aggregates was used to analyze the statistics of the three screening rounds. The aggregates with an average fluorescence intensity >5,000 were counted. Statistical analysis was performed with Gen5™ 3.02 software.

Keima-TDP25 assay analysis: The average fluorescence intensity of Keima-TDP25 aggregates was used to analyze the statistics in the Keima-TDP25 assay. Keima-red with an average fluorescence intensity >1,000 and Keima-green with an average fluorescence intensity >3,000 were counted. Statistical analysis was performed with Gen5™ 3.02 software.

Co-localization analysis: ImageJ was used to analysis the co-localization of fluorescently labeled proteins.

All experiments western blot, micrographs assay, and qPCR assay were carried out at least three independent times with the similar results. Statistical analyses were performed with GraphPad Prism 8. Data were analyzed with a Student’s t-test. Data points are shown as Mean ± SD.

## Results

### Identification of Potent TDP25 Degrader Through High-Throughput Screening

**FIGURE 1:**
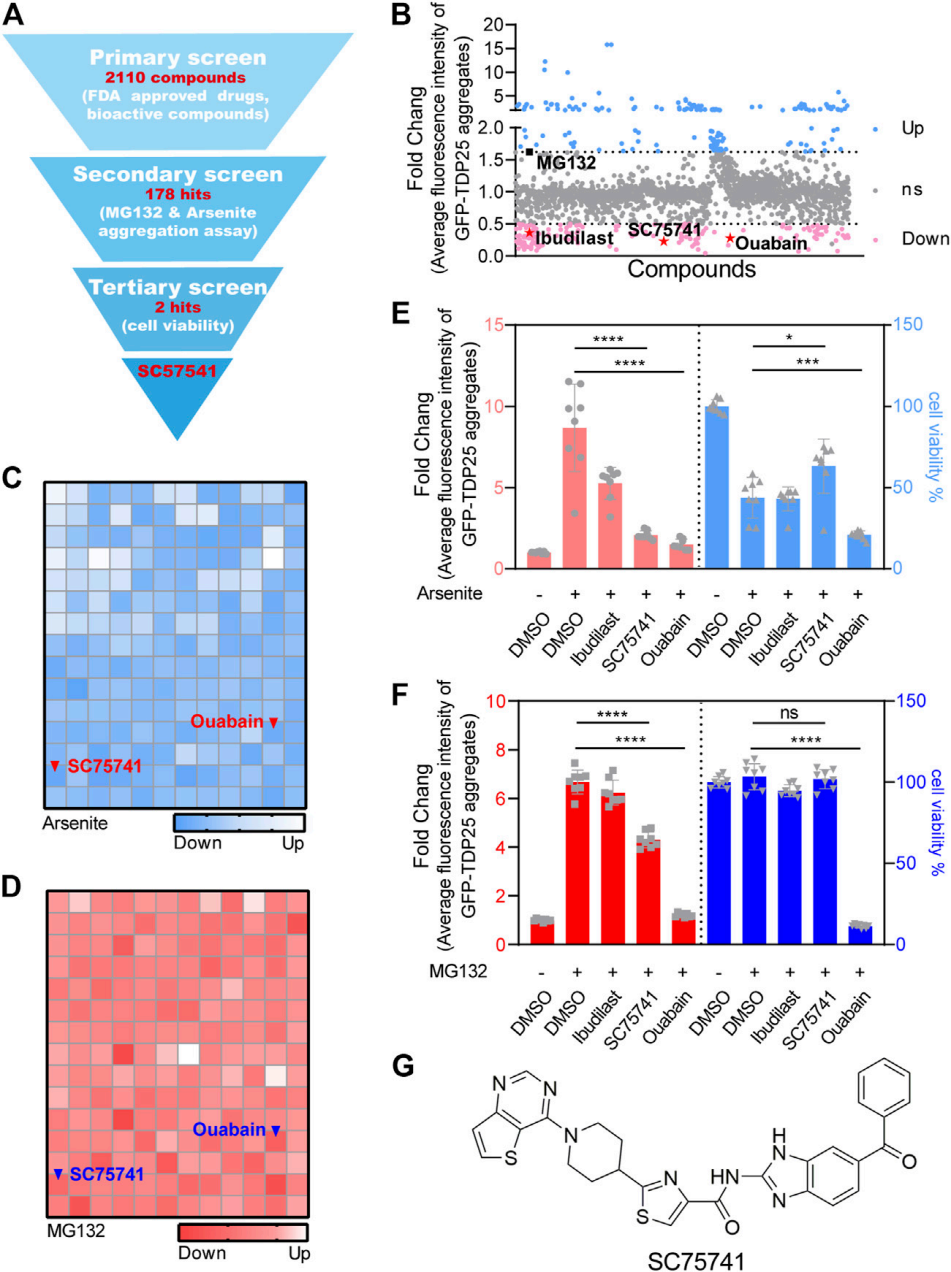
Identification of TDP25 degrader by high-throughput screening. **(A)**. Flowchart of the drug screening and its results. **(B)**. H4GT25 cells were pretreated with 1 μg/mL DOX (doxycycline) for 24 h, incubated with 2110 FDA approved drugs and bioactive compounds for 24 h, and the GFP-TDP25 fluorescence was analyzed by fluorescence microscopy and compared with cells treated with DMSO. The results were presented in the forms of scatter diagram. Skyblue and pink colors represent the degree of increase and decrease of GFP-TDP25 expression, respectively, following drug treatments. Shown in black scatterplot is MG132, and shown in red are ibudilast, ouabain, and SC75741. **(C)**. H4GT25 cells were pretreated with 1 μg/mL DOX for 24 h, treated with arsenite (0.5 mM) for 1 h, incubated with 178 hits for 24 h, and the GFP-TDP25 fluorescence was analyzed by fluorescence microscopy and compared with cells treated with DMSO. The results were presented in the forms of blue heatmap. **(D)**. H4GT25 cells were pretreated with 1 μg/mL DOX for 24 h, treated with MG132 (1 μM) for 12 h, incubated with 178 hits for 24 h, and the GFP-TDP25 fluorescence was analyzed by fluorescence microscopy and compared with cells treated with DMSO. The results were presented in the forms of red heatmap. **(E)**. H4GT25 cells were pretreated with 1 μg/mL DOX for 24 h, treated with arsenite (0.5 mM) for 1 h, and treated with ibudilast (10 μM), SC75741 (10 μM) or ouabain (10 μM) for another 24 h, fluorescence of GFP-TDP25 was analyzed by fluorescence microscopy and Cell viability was determined using CellTiterGlo^®^ assay (data represents Mean ± SD.; *n* = 8, ^****^ *p* < 0.0001, ^***^ *p* < 0.001, ^*^ *p* < 0.05, two-tailed *t* test). **(F)**. H4GT25 cells were pretreated with 1 μg/mL DOX for 24 h, treated with MG132 (1 μM) for 12 h, and treated with ibudilast (10 μM), SC75741 (10 μM) or ouabain (10 μM) for another 24 h, fluorescence of GFP-TDP25 was analyzed by fluorescence microscopy and Cell viability was determined using CellTiterGlo^®^ assay (data represents Mean ± SD.; *n* = 8, ^****^ *p* < 0.0001, two-tailed *t* test). **(G)**. Chemical structure of SC75741.

H4 cell line expressing GFP-TDP25 in a doxycycline (DOX)-inducible manner (H4GT25) was generated to establish a reporter system suitable for high-throughput screening. Ibudilast-treated cells showed a decreased aggregation and were used as positive controls, while MG132-treated cells showed an increased aggregation and were used as negative controls (Supplementary Figure S1A). The screening design is shown in Figure 1A. A total of 2110 FDA-approved drugs and bioactive compounds were screened to identify a potential TDP25 degrader, of which 178 compounds induced a significant decrease in GFP-TDP25 fluorescence by over 50% (Figure 1B). Literature has shown that the number and size of TDP25 aggregates significantly increase after inhibition of proteasome or induction of stress granules (Wolozin, 2012; Chen et al., 2020). Herein, the above two methods were used to perform a second screening with the 178 compounds, of which two candidate compounds (SC75741 and ouabain) decreased GFP-TDP25 (Figures 1C,D). The two compounds were further treated with MG132 or arsenite to determine if they can affect cell viability and confirm whether the decrease of fluorescence is caused by increased cell death. Ouabain induced cell death, while SC75741 did not affect cell viability under both treatments (Figures 1E,F). Live cell imaging further showed that SC75741 significantly reduced the number and size of TDP25 aggregation (Supplementary Figures S1B,C; Supplementary Movie S1). Furthermore, SC75741 treated in H4GT25 or SH-SY5YGT25 cells decreased TDP25 levels in a dose-dependent manner. (Supplementary Figures S1D,E). Moreover, the protein level of TDP25 was substantially reduced after SC75741 treatment in MG132 or arsenite treated H4GT25 cells (Supplementary Figures S1F,G). Importantly, the total lysis, RIPA lysis, and urea lysis were detected using the previously reported method (Winton et al., 2008). Interestingly, SC75741 not only reduced the level of soluble proteins, but also removed insoluble proteins (Supplementary Figure S1H). Therefore, SC75741 was selected for the following study (Figure 1G).

### SC75741 Promotes Clearance of ALS-Related Pathogenic Mutations-Induced Aggregation

**FIGURE 2:**
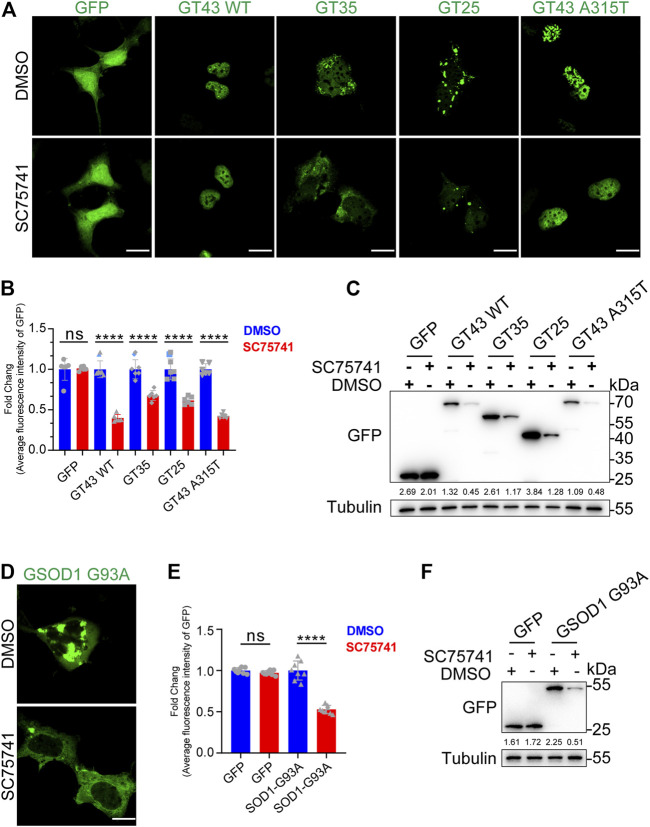
SC75741 increases clearance of ALS-related pathogenic mutations-induced aggregation. **(A)**. SH-SY5Y cells were transfected with indicated plasmids for 24 h, treated with or without 5 μM SC75741 for another 24 h, the GFP fluorescence was done using confocal microscopy. Scale bar, 20 μm. **(B)**. SH-SY5Y cells were transfected with indicated plasmids for 24 h, treated with or without 5 μM SC75741 for another 24 h, the GFP fluorescence was analyzed by fluorescence microscopy. (data represent mean ± SD; *n* = 6, ^****^ *p* < 0.0001, ns, not significant, two-tailed *t* test). **(C)**. SH-SY5Y cells were transfected with indicated plasmids for 24 h, treated with or without 5 μM SC75741 for another 24 h. Cell lysates were immunoblotted with indicated antibodies. The numbers under the blot represent the gray scale quantification (GFP/Tubulin). **(D)**. SH-SY5Y cells were transfected with GFP-SOD1 G93A for 24 h, treated with or without 5 μM SC75741 for another 24 h, the GFP fluorescence was done using confocal microscopy. Scale bar, 20 μm. **(E)**. SH-SY5Y cells were transfected with GFP-SOD1 G93A for 24 h, treated with or without 5 μM SC75741 for another 24 h, the GFP fluorescence was analyzed by fluorescence microscopy. (data represent mean ± SD; *n* = 8, ^****^ *p* < 0.0001, ns, not significant, two-tailed *t* test). **(F)**. SH-SY5Y cells were transfected with GFP-SOD1 G93A for 24 h, treated with or without 5 μM SC75741 for another 24 h. Cell lysates were immunoblotted with indicated antibodies. The numbers under the blot represent the gray scale quantification (GFP/Tubulin).

In the past few decades, over 50 TDP43-related pathological mutations have been identified in ALS patients. The previous experiments have suggested that SC75741 effectively clears TDP25, indicating that SC75741 may have a similar effect on ALS-related TDP43 pathological mutations. In this study, wild-type TDP43, truncated mutation (TDP35, TDP25), and point mutation TDP43 A315T in SH-SY5Y cells were overexpressed to confirm the role of SC75741 on ALS-related TDP43 pathological mutations. The protein levels and GFP fluorescence levels of TDP43-related mutations were partially decreased after SC75741 treatment (Figures 2A–C; Supplementary Figures S2A,B).

SOD1, Cu/Zn superoxide dismutase 1, was the first ALS gene identified in 1993 (Rosen et al., 1993). SOD1 mutations (G93A, G37R, H46R) induce conformational instability, resulting in the formation of intracellular aggregates (Karch et al., 2009). SC75741 effectively cleared SOD1 G93A aggregates (Figures 2D,E), similar to the western blotting experiments (Figure 2F).

Overall, these results indicate that SC75741 reduces the accumulation of various pathological mutations of TDP43 and SOD1.

### SC75741 Inhibits TNF-α-Induced Aggregation Formation and Promotes Aggregation Clearance

**FIGURE 3:**
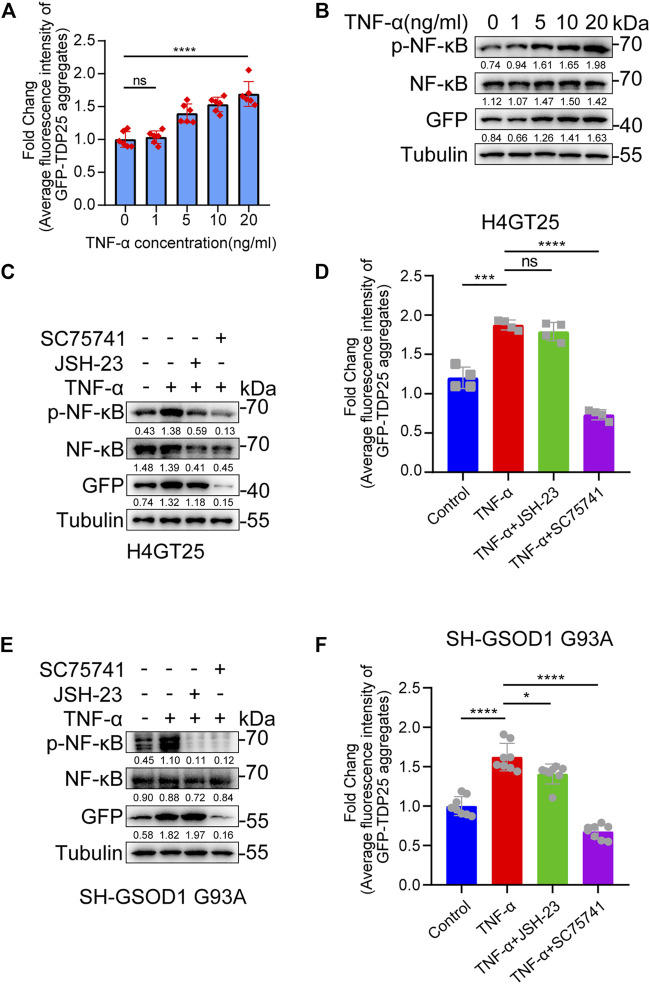
SC75741 inhibits TNF-α-induced aggregation formation and promotes aggregation clearance. **(A)**. H4GT25 cells were pretreated with 1 μg/ml DOX for 24 h, treated with TNF-α (0, 1, 5, 10, and 20 ng/ml) for another 24 h, the GFP fluorescence was analyzed by fluorescence microscopy. (data represent mean ± SD; *n* = 6, ^****^ *p* < 0.0001, ns, not significant, two-tailed *t* test). **(B)**. H4GT25 cells were pretreated with 1 μg/ml DOX for 24 h, treated with TNF-α (0, 1, 5, 10, and 20 ng/ml) for another 24 h. Cell lysates were immunoblotted with indicated antibodies. The numbers under the blot represent the gray scale quantification (GFP/Tubulin, NF-κB/Tubulin, p-NF-κB/Tubulin). **(C)**. H4GT25 cells were pretreated with 1 μg/ml DOX for 24 h, treated with TNF-α (20 ng/ml), JSH-23 (10 μM) or SC75741 (5 μM) for another 24 h. Cell lysates were immunoblotted with indicated antibodies. The numbers under the blot represent the gray scale quantification (GFP/Tubulin, NF-κB/Tubulin, p-NF-κB/Tubulin). **(D)**. H4GT25 cells were pretreated with 1 μg/ml DOX for 24 h, treated with TNF-α (20 ng/ml), DMSO, JSH-23 (10 μM) or SC75741 (5 μM) for another 24 h, the GFP fluorescence was analyzed by fluorescence microscopy. (data represent mean ± SD; *n* = 4, ^****^ *p* < 0.0001, ^***^ *p* < 0.001, ns, not significant, two-tailed *t* test). **(E)**. SH-SY5Y cells were transfected with GFP-SOD1 G93A for 24 h, treated with TNF-α (20 ng/ml), JSH-23 (10 μM) or SC75741 (5 μM) for another 24 h. Cell lysates were immunoblotted with indicated antibodies. The numbers under the blot represent the gray scale quantification (GFP/Tubulin, NF-κB/Tubulin, p-NF-κB/Tubulin). **(F)**. SH-SY5Y cells were transfected with GFP-SOD1 G93A for 24 h, treated with TNF-α (20 ng/ml), JSH-23 (10 μM) or SC75741 (5 μM) for another 24 h, the GFP fluorescence was analyzed by fluorescence microscopy. (data represent mean ± SD; *n* = 6, ^****^ *p* < 0.0001, ^*^ *p* < 0.05, two-tailed *t* test).

Inflammation has been increasingly recognized as an important factor underlying the cell-specific neurodegeneration observed in neurodegenerative diseases, including ALS (Stephenson et al., 2018; Suss et al., 2021). NF-κB, nuclear factor kappa B, is a critical regulator of immune and inflammatory responses (Dresselhaus and Meffert, 2019). Previous studies have shown that the mRNA and protein levels of NF-κB are higher in the spinal cord of ALS patients than in normal patients (Correia et al., 2015). Furthermore, NF-κB inhibitors have been reported to alleviate disease phenotypes in mice overexpressing human TDP43 A315T mutation (Dutta et al., 2017). These studies suggest that inflammation promotes aggregate production in ALS. As expected, we found that TNF-α induced the production of TDP25 aggregates and the level of p-NF-κB in a dose-dependent manner (Figures 3A,B). In addition, by treating TNF-α-induced TDP25 aggregation with NF-κB inhibitor and SC75741 separately, we found that SC75741 not only reduced the level of p-NF-κB but also promoted the clearance of TDP25 aggregates while JSH-23 only reduced the level of p-NF-κB (Figures 3C,D). The same results were obtained on the TNF-α-induced SOD1 G93 A cell model (Figures 3E,F). These results suggest that SC75741 may have another target to promote aggregates clearance.

### c-Abl Is a Potential Target of SC75741

**FIGURE 4:**
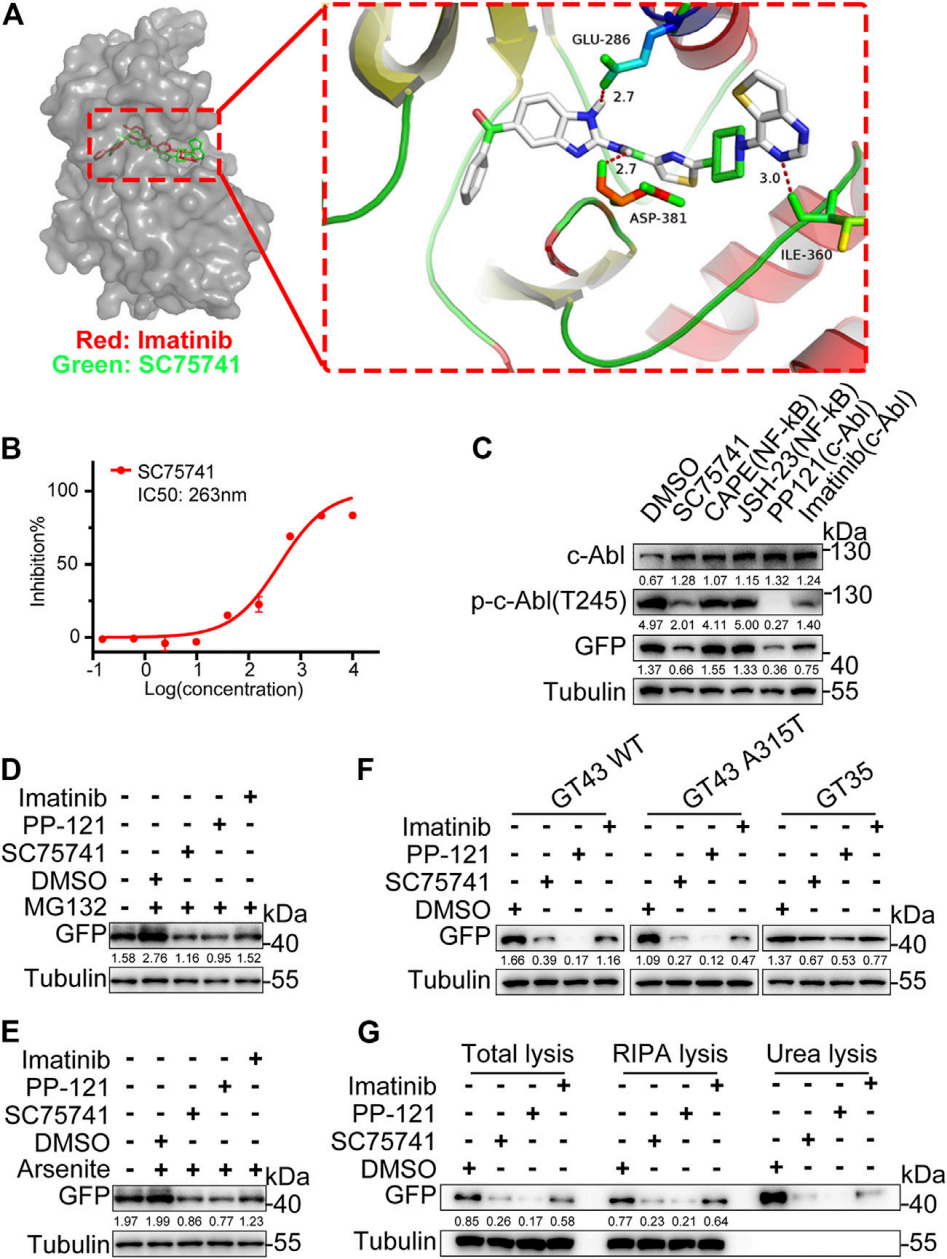
Target identification and validation of SC75741. **(A)**. Molecular docking models of SC75741 with c-Abl (PDB code: 1IEP). The imatinib is shown in red and SC75741 is shown in green. The hydrogen-bonding interactions are shown using red lines. Detailed interactions of SC75741 with c-Abl was amplified as shown in the right. **(B)**. Dose–response curves of c-Abl activity in the presence of SC75741. (data represent from at least two independent experiments). **(C)**. H4GT25 cells were pretreated with 1 μg/ml DOX for 24 h, treated with DMSO, CAPE (10 μM), JSH-23 (10 μM), PP-121 (5 μM), imatinib (5 μM) or SC75741 (5 μM) for another 24 h. Cell lysates were immunoblotted with indicated antibodies. The numbers under the blot represent the gray scale quantification (GFP/Tubulin, p-c-Abl(T245)/Tubulin, c-Abl/Tubulin). **(D)**. H4GT25 cells were pretreated with 1 μg/ml DOX for 24 h, treated with 1 μM MG132 for 12 h, treated with DMSO, PP-121 (5 μM), imatinib (5 μM) or SC75741 (5 μM) for another 24 h. Cell lysates were immunoblotted with indicated antibodies. The numbers under the blot represent the gray scale quantification (GFP/Tubulin). **(E)**. H4GT25 cells were pretreated with 1 μg/mL DOX for 24 h, treated with 0.5 mM arsenite for 1 h, treated with DMSO, PP121 (5 μM), imatinib (5 μM) or SC75741 (5 μM) for another 24 h. Cell lysates were immunoblotted with indicated antibodies. The numbers under the blot represent the gray scale quantification (GFP/Tubulin). **(F)**. SH-SY5Y cells were transfected with indicated plasmids for 24 h, treated with DMSO, PP121 (5 μM), imatinib (5 μM) or SC75741 (5 μM) for another 24 h, Cell lysates were immunoblotted with indicated antibodies. The numbers under the blot represent the gray scale quantification (GFP/Tubulin). **(G)**. H4GT25 cells were pretreated with 1 μg/ml DOX for 24 h, treated with DMSO, PP-121 (5 μM), imatinib (5 μM) or SC75741 (5 μM) for another 24 h, total lysates, RIPA lysates and urea lysates were immunoblotted with indicated antibodies. The numbers under the blot represent the gray scale quantification (GFP/Tubulin).

SC75741 is an NF-κB inhibitor (Ehrhardt et al., 2013). NF-κB inhibitors (JSH-23, Caffeic acid phenethyl ester) failed to decrease the protein level of TDP25, indicating that the NF-κB pathway is not the main factor in this degradation (Figure 4C). Some reports have shown that the structure of benzimidazolamide can inhibit the activity of c-Abl (Hendrick et al., 2016). SC75741 contains the benzimidazolamide framework, suggesting that c-Abl is the potential target of SC75741. The modeled complex structures of the c-Abl kinase domain with SC75741 and imatinib are shown in Figure 4A. SC75741 binds to the activation loop of c-Abl, forming hydrogen bond interactions with the surrounding carbonyl groups of Glu-296, Asp-381, and Ile-360. Furthermore, *in vitro* kinase assay was conducted to obtain an inhibitory concentration (IC50) of 263 nM for further analysis (Figure 4B). Other c-Abl inhibitors (PP-121, imatinib) also reduced the aggregation of TDP25 induced *via* MG132 and arsenite treatments (Figures 4D,E). Meanwhile, c-Abl inhibitors also reduce the protein levels of various pathological mutations of TDP43 (Figure 4F). In this study, c-Abl inhibitors degraded both the soluble TDP25 and the insoluble TDP25 aggregates (Figure 4G). Therefore, SC75741 promotes the elimination of TDP25 and TDP43-related aggregates by inhibiting c-Abl.

### SC75741 Enhances Autophagy *via* the ATG5 Pathway

**FIGURE 5:**
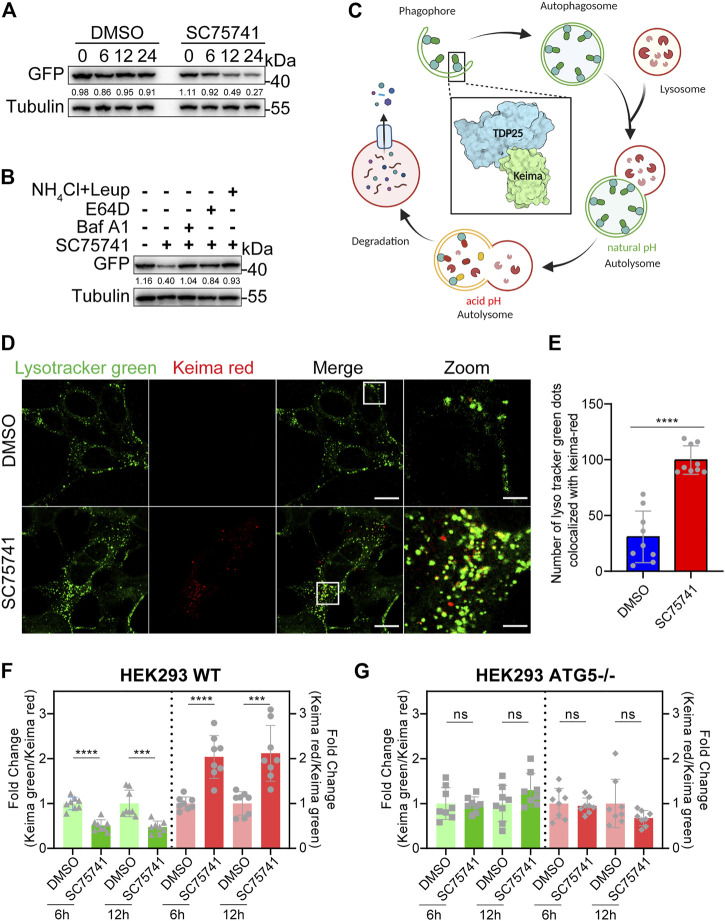
SC75741 enhances autophagy via ATG5 pathway. **(A)**. H4GT25 cells were pretreated with 1 μg/ml DOX for 24 h, wash out DOX, treated with DMSO or 5 μM SC75741 for 6,12, and 24 h. Cell lysates were immunoblotted with indicated antibodies. The numbers under the blot represent the gray scale quantification (GFP/Tubulin). **(B)**. H4GT25 cells were pretreated with 1 μg/ml DOX for 24 h, treated with 5 μM SC75741 with or without, Bafilomycin A1 (100 nM), NH4Cl (20 mM), Leupeptin (100 nM), E-64D (10 μM) for 8 h. Cell lysates were immunoblotted with indicated antibodies. The numbers under the blot represent the gray scale quantification (GFP/Tubulin). **(C)**. Schematic model of the detection of autophagy with Keima-TDP25 overexpressed in SH-SY5Y cells. The graphical was created using BioRender.com. **(D)**. SH-SY5YKT25 cells were pretreated with 1 μg/ml DOX for 24 h, treated with or without 5 μM SC75741 for 3 h and the co-localization between Keima-red and LysoTracker green was done using live cell imaging and confocal microscopy. Scale bar, 20 μm; insets: scale bar, 5 μm. **(E)**. SH-SY5YKT25 cells were pretreated with 1 μg/ml DOX for 24 h, treated with or without 5 μM SC75741 for 3 h and the co-localization between Keima-red and LysoTracker green was analyzed by two-tailed t test. (data represent mean ± SD; n = 9, ^****^ *p* < 0.0001). **(F)**. HEK293 WT cells were transfected with Keima-TDP25, pretreated with 1 μg/ml DOX for 24 h, treated with DMSO or 5 μM SC75741 for 6 and 12 h, and the Keima-TDP25 fluorescence was analyzed by fluorescence microscopy and compared with cells treated with DMSO. (data represent mean ± SD; n = 8, ^****^ *p* < 0.0001, ^***^ *p* < 0.001). **(G)**. HEK293 ATG5-/- cells were transfected with Keima-TDP25, pretreated with 1 μg/ml DOX for 24 h, treated with DMSO or 5 μM SC75741 for 6 and 12 h, and the Keima-TDP25 fluorescence was analyzed by fluorescence microscopy and compared with cells treated with DMSO. (data represent mean ± SD; n = 8, ns, not significant, two-tailed *t* test).

This study investigated whether SC75741 down-regulates protein by inhibiting synthesis or promoting degradation. SC75741 significantly promoted TDP25 degradation when doxycycline was removed to block protein expression (Figure 5A). TDP25 degradation, induced by SC75741, was blocked by lysosome inhibitors (E64D, bafilomycin A1 or NH4Cl/Leupeptin) indicated that the degradation was through lysosome pathway (Figure 5B). Meanwhile, E64D and bafilomycin A1 were able to increase the levels of LC3-II, and SC75741 further increase it, these results indicate that SC75741 may increase the autophagic flux (Supplementary Figure S3D).

Keima, a coral-derived fluorescent protein, is a red fluorescent signal (586 nm) at acidic pH and a green fluorescent signal (438 nm) at neutral pH. We generated a stable cell line, which overexpressed Keima-TDP25 in SH-SY5Y (Figure 5C). The Keima showed a red fluorescence when TDP25 entered the lysosome for degradation, confirming that SC75741 treatment enhances the Keima-Red fluorescence and lysosome colocalization (Figures 5D,E). The Keima-Green/Keima-red ratio was normalized to the degree of TDP25 degradation, and Keima-red/Keima-green ratio was used as a measure for lysosome transfer. Our results showed that SC75741 reduced the levels of TDP25 by enhancing lysosome degradation (Supplementary Figures S3A,B).

ATG5, autophagy-related 5, is essential for autophagosome vesicle formation. Knocking down or knocking out ATG5 downregulates autophagy, suggesting that ATG5 influences autophagy (Mizushima et al., 2001; Suzuki et al., 2001). The HEK293 WT and ATG5^−/−^ cell lines overexpressing Keima-TDP25 showed that the decreased ratio of Keima-green/Keima-red under SC75741 treatment was ATG5 dependent (Figures 5F,G), similar to western blotting experiment (Supplementary Figure S3C). Taken together, these findings indicate that the SC75741-induced TDP25 degradation is ATG5-dependent.

### SC75741 Activates TFEB Independently of mTORC1 Activity

**FIGURE 6:**
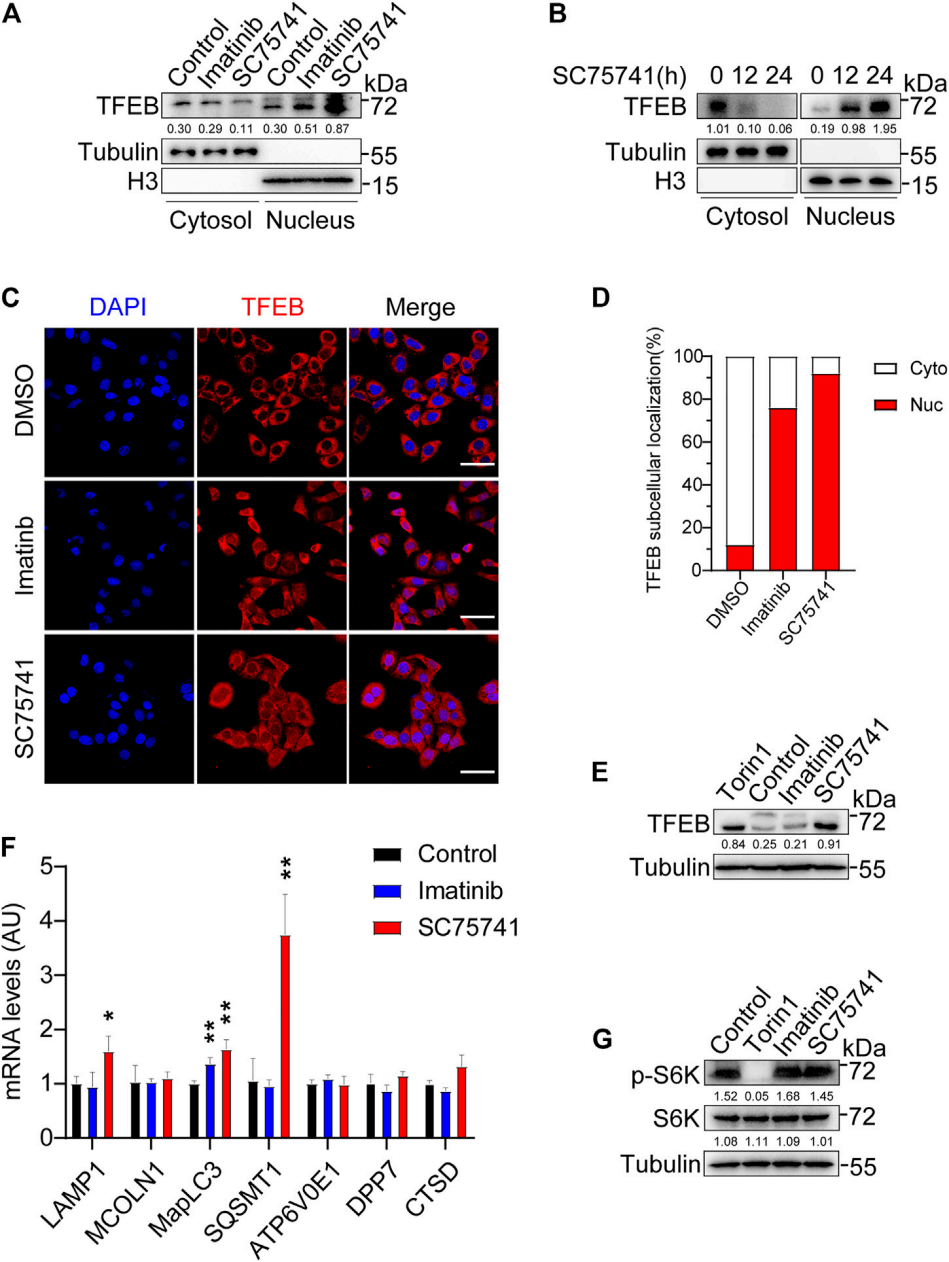
SC75741 activates TFEB independently of mTORC1 activity. **(A)**. H4GT25 cells were pretreated with 1 μg/ml DOX for 24 h, treated with DMSO, imatinib (5 μM) or SC75741 (5 μM) for another 24 h. Nuclear lysates and cytoplasmic lysates were immunoblotted with indicated antibodies. The numbers under the blot represent the gray scale quantification (TFEB/Tubulin, TFEB/H3). **(B)**. H4GT25 cells were pretreated with 1 µg/mL DOX for 24h, treated with SC75741 (5 μM) for 12 and 24 h. Nuclear lysates and cytoplasmic lysates were immunoblotted with indicated antibodies. The numbers under the blot represent the gray scale quantification (TFEB/Tubulin, TFEB/H3). **(C)**. H4GT25 cells were pretreated with 1 μg/ml DOX for 24 h, treated with DMSO, imatinib (5 μM) or SC75741 (5 μM) for another 12h, and then the cells were subjected to immuno-fluorescence analysis using an anti-TFEB antibody. Scale bar, 40 μm. **(D)**. Quantification of the percentage of TFEB-positive cells in the nucleus and TFEB-positive cells in the cytoplasm. (data represents mean ± SD; DMSO (*n* =110 cells), imatinib (*n* =112 cells), SC75741 (*n* =101 cells)). **(E)**. H4GT25 cells were pretreated with 1 μg/ml DOX for 24 h, treated with DMSO, Torin1 (1 μM), imatinib (5 μM) or SC75741 (5 μM) for another 12 h. Cell lysates were immunoblotted with indicated antibodies. The numbers under the blot represent the gray scale quantification (TFEB/Tubulin). **(F)**. q-PCR analysis of different mRNA TFEB target genes in H4GT25 cells treated with imatinib (5 μM) and SC75741 (5 μM) for 12 h. **(G)**. H4GT25 cells were pretreated with 1 μg/ml DOX for 24 h, treated with DMSO, Torin1 (1 μM), imatinib (5 μM) or SC75741 (5 μM) for another 12 h. Cell lysates were immunoblotted with indicated antibodies. The numbers under the blot represent the gray scale quantification (p-S6K/Tubulin, S6K/Tubulin,).

TFEB, transcription factor EB, which positively regulates the expression autophagy-related genes to induce autophagosome formation, autophagosome-lysosome fusion, and degradation of autophagy substrates (Martini-Stoica et al., 2016). Prior studies have found that the tyrosine kinase c-Abl regulates TFEB nuclear translocation (Contreras et al., 2020) which suggesting that SC75741 may also has the similar function. As expected, we found that the treatment of SC75741 strikingly enhanced TFEB nuclear translocation in a time-dependent manner (Figures 6A–D). It has been shown that when TFEB enters the nucleus, it is accompanied by electrophoretic shift phenomenon which is due to its de-phosphorylation. Interestingly, SC75741, which promoted TFEB nuclear translocation, also induced an electrophoretic mobility of the endogenous TFEB compared with the control condition (Figure 6E). Notably, SC75741 increased the expression of several TFEB target genes such as MapLC3, SQSMT1, and LAMP1 in H4GT25 cells (Figure 6F). These results suggest that p62, lamp1 and LC3 might be involved in TDP25 aggregates degradation pathway.

It is well known that the regulation of TFEB nuclear localization mediated by mTORC1 (Settembre et al., 2012). Previous studies have shown that inhibition of c-Abl induced TFEB nuclear translocation is independent on mTORC1 (Contreras et al., 2020). To further analyze whether TFEB nuclear translocation induced by SC75741 is independent on mTORC1, we measured the phosphorylation of p70 S6 kinase (phospho-S6K). As shown in Figure 6G, the level of phosphorylated p70S6K is not affected after SC75741 and imatinib treatment. To this end, we demonstrated that SC75741 could increase TFEB nuclear translocation by an mTORC1-independent TFEB regulatory pathway mediated by the c-Abl.

### SC75741-Induced TDP25 Degradation is p62 and LC3C Dependent

**FIGURE 7:**
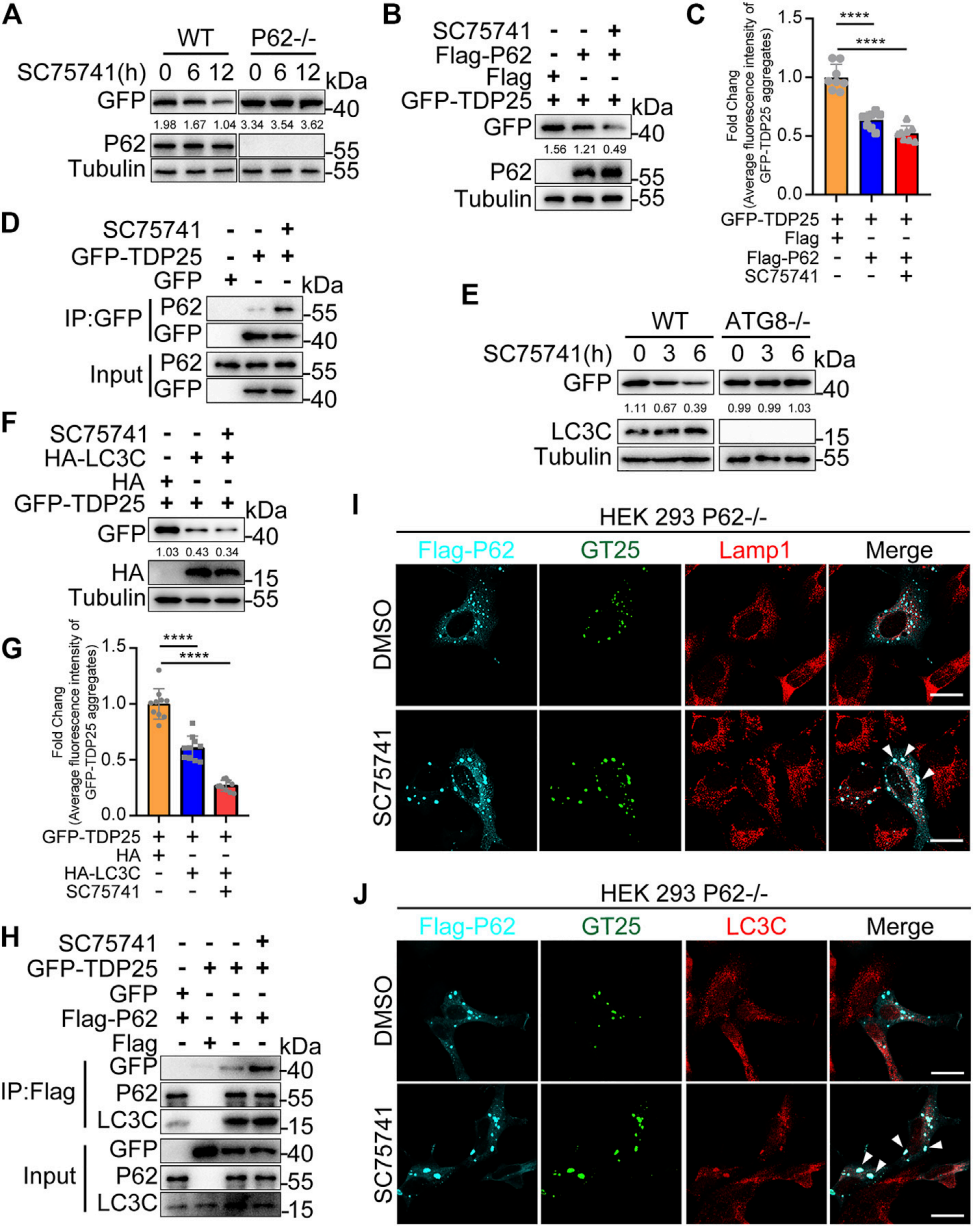
SC75741-induced TDP25 degradation depends on p62 and LC3C. **(A)**. HEK293 WT and p62-/- cells were transfected with GFP-TDP25 for 24 h, treated with SC75741 (5 μM) for 12 and 24 h. Cell lysates were immunoblotted with indicated antibodies. The numbers under the blot represent the gray scale quantification (GFP/Tubulin). **(B)**. HEK293 p62-/- cells were transfected with GFP-TDP25 and Flag-p62 for 24 h, treated with SC75741 (5 μM) for 12 h. Cell lysates were immunoblotted with indicated antibodies. The numbers under the blot represent the gray scale quantification (GFP/Tubulin). **(C)**. HEK293 p62-/- cells were transfected with GFP-TDP25 and Flag-p62 for 24 h, treated with SC75741 (5 μM) for 12 h. The GFP fluorescence was analyzed by fluorescence microscopy. (data represent mean ± SD; n = 8, ^****^ *p* < 0.0001, two-tailed t test). **(D)**. HEK293 WT cells were transfected with GFP-TDP25 for 24 h, treated with or without SC75741 (5 μM) for another 6 h, the interaction between p62 and TDP25 was analyzed by immunoprecipitation. **(E)**. Hela WT and ATG8-/- cells were transfected with GFP-TDP25 for 24 , treated with SC75741 (5 μM) for 3 and 6 . Cell lysates were immunoblotted with indicated antibodies. The numbers under the blot represent the gray scale quantification (GFP/Tubulin). **(F)**.Hela ATG8-/- cells were transfected with GFP-TDP25 and HA-LC3C for 24 h, treated with SC75741 (5 μM) for 6 h. Cell lysates were immunoblotted with indicated antibodies. The numbers under the blot represent the gray scale quantification (GFP/Tubulin). **(G)**. Hela ATG8-/- cells were transfected with GFP-TDP25 and HA-LC3C for 24 h, treated with SC75741 (5 μM) for 6 h. The GFP fluorescence was analyzed by fluorescence microscopy. (data represent mean ± SD; n = 10, ^****^ *p* < 0.0001, two-tailed t test). **(H)**. HEK293 p62-/- cells were transfected with GFP, GFP-TDP25, Flag and Flag-p62 for 24 h, treated with or without SC75741 (5 μM) for another 6 h, the interaction of p62 with TDP25 and LC3C were analyzed by immunoprecipitation. **(I)**. HEK293 p62-/- cells were transfected with GFP-TDP25 and Flag-p62 for 24 h, treated with SC75741 (5 μM) for 6 h and the co-localization among TDP25, p62, lamp1 were done using immunofluorescence and confocal microscopy. Arrows indicate the co-localization. Scale bar, 20 μm. **(J)**. HEK293 p62-/- cells were transfected with GFP-TDP25 and Flag-p62 for 24 h, treated with SC75741 (5 μM) for 6h and the co-localization among TDP25, p62, LC3C were done using immunofluorescence and confocal microscopy. Arrows indicate the co-localization. Scale bar, 20 μm.

P62/SQSTM1, an autophagy adaptor protein, can bind ubiquitinated substrates or protein aggregates for degradation *via* autophagy, thus protecting the cytosol from the toxic effects of misfolded proteins (Lippai and Low, 2014). In this study, we examined whether p62, as an autophagy adaptor, is involved in the autophagic degradation of TDP25 after SC75741 treatment. The results showed that TDP25 degradation after SC75741 treatment was p62 dependent in p62 knockout HEK293T cells (Figures 7A–C; Supplementary Figure S4A). Moreover, pull-down experiments showed that SC75741 increased the interaction between p62 and TDP25 (Figure 7D). These findings indicate that SC75741 promotes the clearance of TDP25 in a p62 dependent manner.

LC3, microtubule-associated protein 1 light-chain 3, is essential for elongation and maturation of the autophagosome and is similar to yeast ATG8 (Kabeya et al., 2000; Lee and Lee, 2016). Co-immunoprecipitation assays were used to assess the interaction between TDP25 and LC3 to investigate whether LC3 is involved in SC75741-induced TDP25 degradation. The interaction between LC3C and GFP-TDP25 was enhanced after SC75741 treatment (Supplementary Figure S4B). TDP25 degradation, induced by SC75741, was significantly blocked in ATG8KO cells (Figure 7E). However, TDP25 degradation was restored after re-overexpressing LC3C in ATG8KO cells (Figures 7F,G). We also evaluated whether p62 is an intermediate adapter protein in the interaction between LC3C and TDP25. SC75741 treatment also promoted the interaction between p62 with LC3C or TDP25 (Figure 7H). These results were also confirmed by immunofluorescence in p62KO cells, which overexpressed p62 and TDP25 (Figure 7J, Supplementary Figure S4D). Besides, SC75741 treatment enhanced colocalization of Lamp1 and TDP25 (Figure 7I, Supplementary Figure S4C).

Taken together, these results indicate that SC75741 can promote the interaction of p62 with LC3C and TDP25, thus facilitating TDP25 degradation through the autophagy pathway.

## Discussion and Conclusion

Abnormal aggregation of pathological TDP43 is associated with neurodegenerative diseases, including amyotrophic lateral sclerosis (ALS), frontotemporal dementia (FTD), and Alzheimer’s disease (AD). TDP25, a C-terminal fragment of TDP43, is a key aggregate component found in the brains of ALS patients. Our study showed that SC75741, as a novel multi-target drug, promotes TDP25 degradation or misfolded TDP43-related proteins through the autophagy pathway. C-Abl, as a new target of SC75741, is essential in enhancing TFEB nuclear translocation by an mTORC1-independent TFEB regulatory pathway to promote TDP25 degradation. SC75741, as an intermediate, promotes the interaction between p62 with LC3 and TDP25, facilitating TDP25 degradation.

Our study focused on TDP25, which has the highest degree of pathological aggregation, and a cell-based image high-throughput screening assay was conducted. HSP90, PI3K, Akt, mTOR, and MAPK inhibitors reduced the TDP25 levels in the first screening, consistent with the previous reports (Jinwal et al., 2012; Xia et al., 2016; Li et al., 2017), showing that the screening system was functional and reliable. Interestingly, some inhibitors, such as the proteasome, apparent recognition protein domain, microtubule-associated, and topoisomerase inhibitors, promoted TDP25 expression, indicating that the impairment or mutation of these target proteins can cause ALS. MG132, a proteasome inhibitor, was used in the second screening by blocking the proteasome pathway to increase aggregate production. These results suggested that the candidate molecules screened by the above method could be through the lysosomal pathway. Moreover, the final target compound was screened through cell viability experiments. SC75741, as a target molecule, promoted TDP25 degradation through the lysosomal pathway. SC75741 also showed a cytoprotective effect.

Increasing evidence suggests that c-Abl plays important roles in neurodegenerative diseases. *In vitro* studies show that c-Abl directly interacts with α-synuclein and catalyzes its phosphorylation at tyrosine 39 (pY39) (Mahul-Mellier et al., 2014). Furthermore, the c-Abl-mediated phosphorylation of α-synuclein can cause the destruction of normal protein degradation pathways. Recent evidence suggests that c-Abl phosphorylates tau protein and promotes neurofibrillary tangles to form aggregates in AD (Estrada et al., 2011). Our study found that c- Abl inhibitors degrade ALS-related disease proteins by promoting autophagy. It is unclear whether these ALS-related pathogenic proteins are the substrates of c-Abl and whether the inhibition of kinase activity will affect the modification of downstream substrates. Further studies are needed to explore the molecular mechanisms underlying the role of c-Abl in regulating the aggregation of TDP43 and to validate c-Abl as a target for the treatment of ALS-related pathogenic mutations.

Past studies have found that the mRNA and protein levels of NF-κB are higher in the spinal cord of ALS patients than in normal patients (Correia et al., 2015). Furthermore, NF-κB inhibitor alleviates disease phenotypes has been experimentally verified in mice overexpressing human TDP43 A315T mutation (Dutta et al., 2017). Surprisingly, our study demonstrated that SC75741 eliminates TDP43-related mutant protein *via* the inhibition of c-Abl and the NF-κB pathway. Ibudilast, an anti-inflammatory and neuroprotective oral agent, is currently at clinical trials to treat progressive multiple sclerosis and ALS. These findings suggest that inflammation also promotes ALS occurrence. However, how inflammation affects the formation of TDP25 aggregates and how the formation of TDP25 aggregates promotes inflammation is unclear. SC75741, as a multi-target drug, could be a promising strategy for treating ALS by inhibiting inflammation and promoting aggregate degradation.

In conclusion, our study demonstrates for the first time that SC75741 as a novel autophagy enhancer can inhibit c-Abl pathway, activate autophagy, and trigger TDP43-related aggregates clearance by the ALP. Our findings will facilitate the development of dual-targeted inhibition of c-Abl and NF-κB as a promising drug target for the treatment of ALS.

## Data Availability

The original contributions presented in the study are included in the article/Supplementary Material, further inquiries can be directed to the corresponding authors.
