# Supplementary material for: SC75741, A Novel c-Abl Inhibitor, Promotes the Clearance of TDP25 Aggregates via ATG5-Dependent Autophagy Pathway
Source: Front Pharmacol. 2021 Oct 29;12:741219. doi: 10.3389/fphar.2021.741219 (PMC8586708; doi:10.3389/fphar.2021.741219)
Supplement: Supplementary file 1 [file DataSheet1.pdf]

Supplementary Material

Supplementary Figures

Figure S1

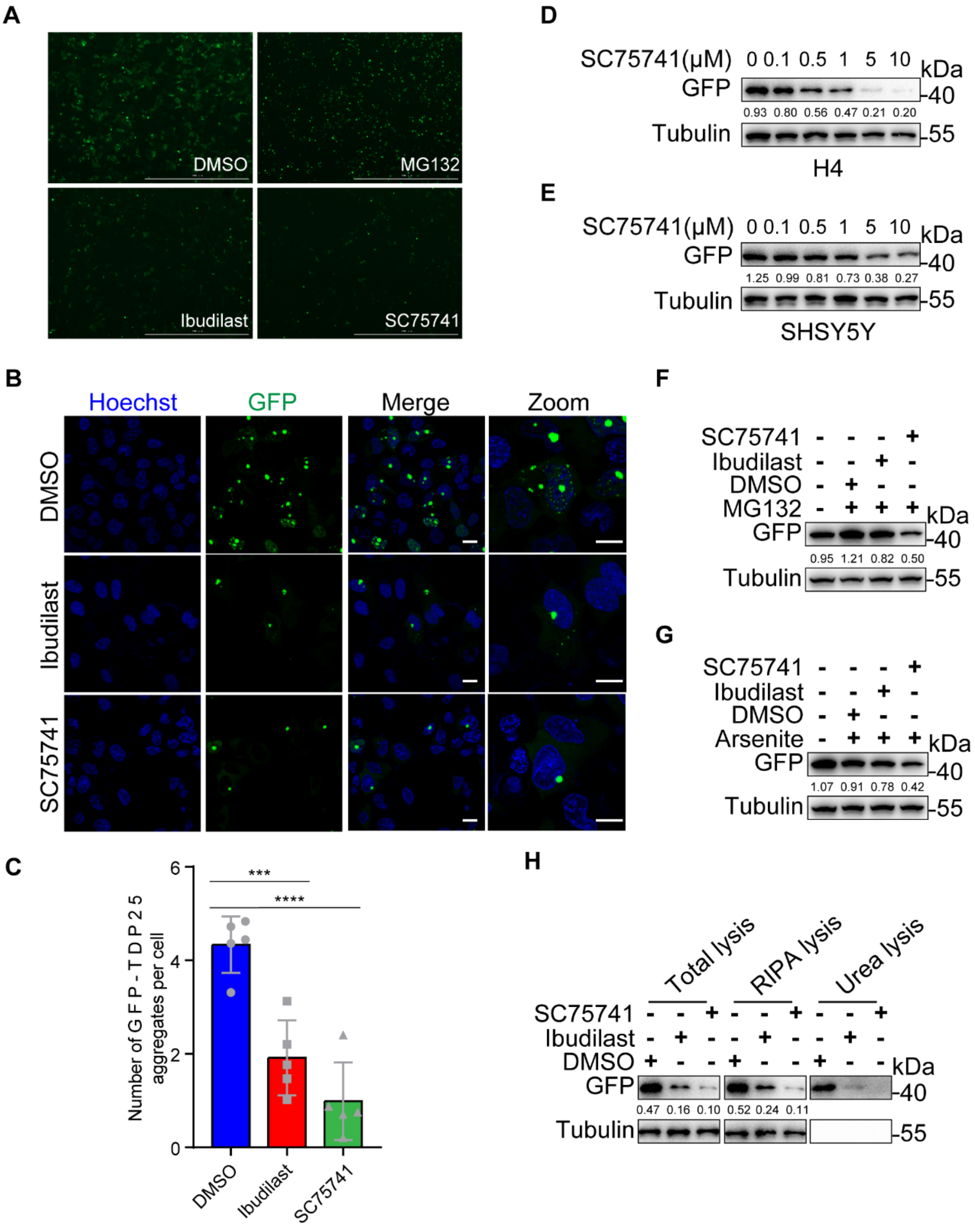

Figure S1. Identification of SC75741 as a TDP25 degradator

A. High-throughput screening image, MG132 is shown as a negative control, Ibudilast is shown as a positive control, SC75741 is shown as a candidate agent.

Scale bar, 1000 μm;

B. H4GT25 cells were pretreated with 1 μg/mL DOX for 24h, treated with DMSO, ibudilast (10 μM) or SC75741 (5 μM) for another 24h, live cell imaging was performed with confocal microscopy. Scale bar, 20 μm

C. H4GT25 cells were pretreated with 1 μg/mL DOX for 24h, treated with DMSO, ibudilast (10 μM) or SC75741 (5 μM) for another 24h, live cell imaging was performed with confocal microscopy. Quantification of the aggregates per cell (data represents mean ± SD; DMSO (n = 190 cells), Ibudilast (n = 336 cells), SC75741 (n = 233 cells), ****$p < 0.0001$, ***$p < 0.001$, two-tailed t test).

D. H4GT25 cells were pretreated with 1 μg/mL DOX for 24h, treated with SC75741(0 μM, 0.1 μM, 0.5 μM, 1 μM, 5 μM and 10 μM) for another 24h. Cell lysates were immunoblotted with indicated antibodies. The numbers under the blot represent the gray scale quantification (GFP/Tubulin).

E. SH-SY5Y-GT25 cells were pretreated with 1 μg/mL DOX for 24h, treated with SC75741(0 μM, 0.1 μM, 0.5 μM, 1 μM, 5 μM and 10 μM) for another 24h. Cell lysates were immunoblotted with indicated antibodies. The numbers under the blot represent the gray scale quantification (GFP/Tubulin).

F. H4GT25 cells were pretreated with 1 μg/mL DOX for 24h, treated with 1 μM MG132 for 12h, along with DMSO, ibudilast (10 μM) or SC75741 (5 μM) for 24h. Cell lysates were immunoblotted with indicated antibodies. The numbers under the blot represent the gray scale quantification (GFP/Tubulin).

G. H4GT25 cells were pretreated with 1 μg/mL DOX for 24h, treated with 0.5 mM arsenite for 1h, along with DMSO, ibudilast (10 μM) or SC75741 (5 μM) for 24h. Cell lysates were immunoblotted with indicated antibodies. The numbers under the blot represent the gray scale quantification (GFP/Tubulin).

H. H4GT25 cells were pretreated with 1 μg/mL DOX for 24h, treated with or without 5 μM SC75741 for another 24h, total lysates, RIPA lysates and urea lysates were immunoblotted with indicated antibodies. The numbers under the blot represent the gray scale quantification (GFP/Tubulin).

Figure S2

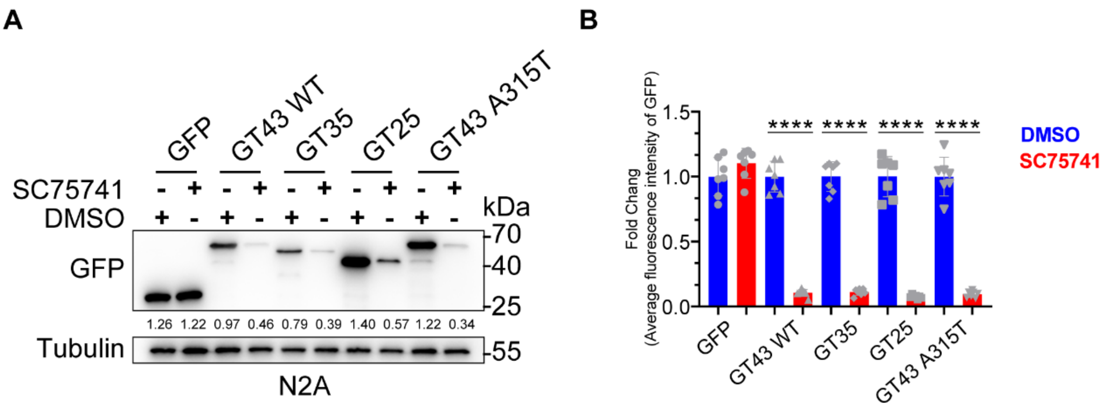

Figure S2. SC75741 increases clearance of ALS-related pathogenic mutations-

induced aggregation in animal cells

A. N2A cells were transfected with indicated plasmids for 24h, treated with or without 5 μM SC75741 for another 24h. Cell lysates were immunoblotted with indicated antibodies. The numbers under the blot represent the gray scale quantification (GFP/Tubulin).

B. N2A cells were transfected with indicated plasmids for 24h, treated with or without 5 μM SC75741 for another 24h, the GFP fluorescence was analyzed by fluorescence microscopy. (data represent mean ± SD; n = 6, ****$p < 0.0001$, two-tailed *t* test).

Figure S3

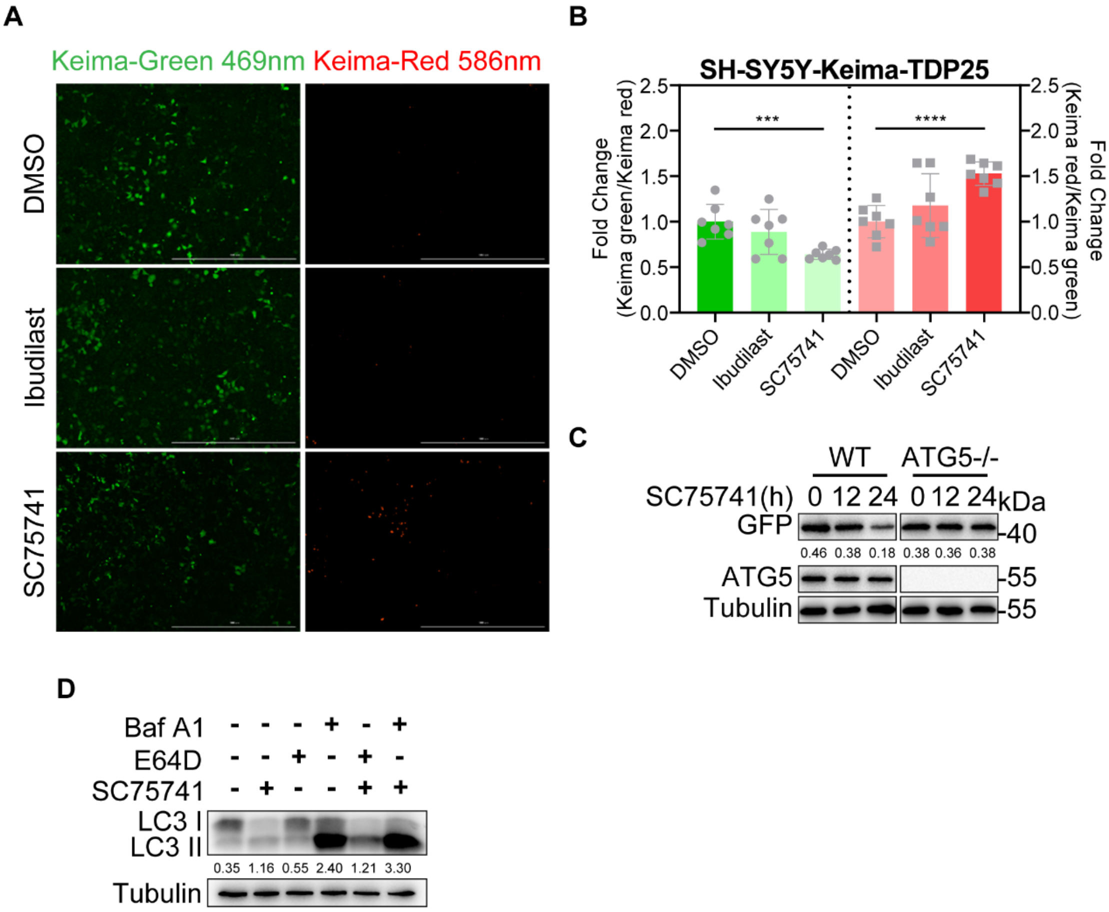

Figure S3. SC75741 enhances autophagy via ATG5 pathway in Keima-TDP25 assays

A. SH-SY5YKT25 cells were pretreated with 1 μg/mL DOX for 24h, treated with DMSO, ibudilast (10 μM) or SC75741 (5 μM) for another 24h, and the Keima-TDP25 fluorescence was analyzed by fluorescence microscopy.

B. SH-SY5YKT25 cells were pretreated with 1 μg/mL DOX for 24h, treated with DMSO, ibudilast (10 μM) or SC75741 (5 μM) for another 24h, and the Keima-TDP25 fluorescence was analyzed by fluorescence microscopy and compared with

cells treated with DMSO. (data represent mean ± SD.; n = 7, ****p < 0.0001, ***p < 0.001, two-tailed *t* test).

C. HEK293 WT and ATG5-/- cells were transfected with GFP-TDP25 for 24h, treated with SC75741 (5 μM) for 12h and 24h. Cell lysates were immunoblotted with indicated antibodies. The numbers under the blot represent the gray scale quantification (GFP/Tubulin).

D. HEK293 WT cells were treated with 5 μM SC75741 with or without, Bafilomycin A1 (100 nM), E-64D (10 μM) for 5h. Cell lysates were immunoblotted with indicated antibodies. The numbers under the blot represent the gray scale quantification (LC3 II/LC3 I).

Figure S4

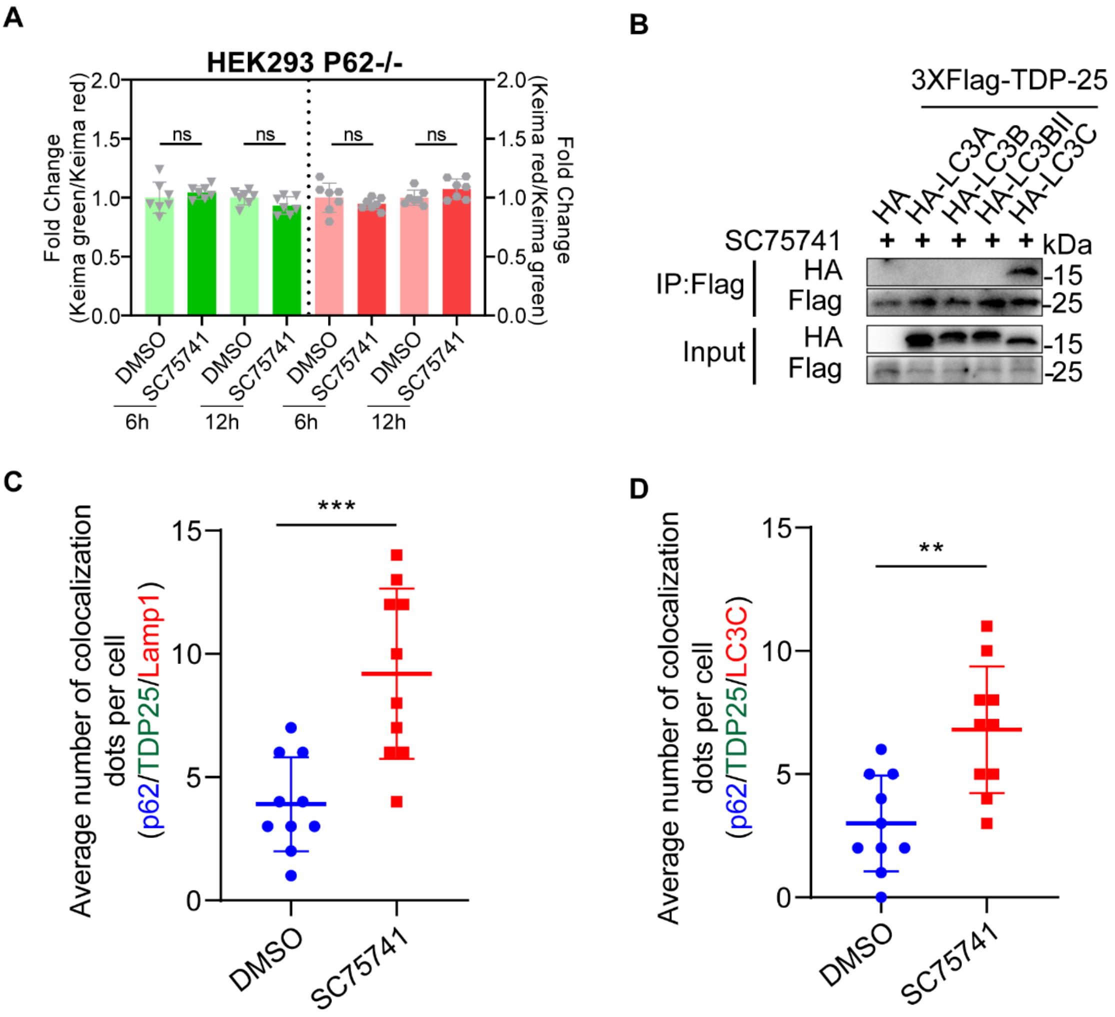

Figure S4. SC75741-induced TDP25 degradation depends on p62 and LC3C

A. HEK293 P62-/- cells were transfected with Keima-TDP25, pretreated with 1 μg/mL DOX for 24 h, treated with DMSO or 5 μM SC75741 for 6h and 12h, and the Keima-TDP25 fluorescence was analyzed by fluorescence microscopy and

compared with cells treated with DMSO. (data represent mean ± SD; n = 7, ns, not significant, two-tailed t test).

B. HEK293 WT cells were transfected with Flag-TDP25 and HA-tagged ATG8 family proteins for 24h, treated with or without SC75741 (5 μM) for another 6h, the interaction between ATG8 family proteins and TDP25 were analyzed by immunoprecipitation.

C. Quantification of the average number of colocalization among TDP25, p62, lamp1 in cells were analyzed by two-tailed t test. (data represent mean ± SD; DMSO (n = 10 cells), SC75741 (n = 10 cells), ***p < 0.001, two-tailed t test).

D. Quantification of the average number of colocalization among TDP25, p62, LC3C in cells were analyzed by two-tailed t test. (data represent mean ± SD; DMSO (n = 10 cells), SC75741 (n = 10 cells), **p < 0.01, two-tailed t test).
